# P-128. Analysis of ARV Blood-Brain Barrier Penetration and Expression of Transporters in Human Astrocytes

**DOI:** 10.1093/ofid/ofaf695.355

**Published:** 2026-01-11

**Authors:** Johid Malik, Nathaniel J Rhodes, Ukamaka O Modebelu, Tim Mykris, Lee Winchester, Courtney Fletcher, Sean Avedissian

**Affiliations:** UNMC, omaha, Nebraska; Midwestern University, Downers Grove, IL; UNMC, omaha, Nebraska; UNMC, omaha, Nebraska; UNMC, omaha, Nebraska; University of Nebraska, Omaha, Nebraska; University of Nebraska Medical Center, Omaha, NE

## Abstract

**Background:**

Antiretrovirals (ARVs) demonstrate limited penetration across the blood brain barrier (BBB) and serum protein binding of ARVs influences BBB penetration. The expression level of ATP-binding cassette (ABC) transporters in human astrocytes is unknown despite being an integral part of the BBB and regulating CNS concentrations of ARVs. We evaluated BBB penetration of eight ARVs in a novel 4-cell BBB model and performed protein expression analysis for ABC transporter expression.

**Figure:**
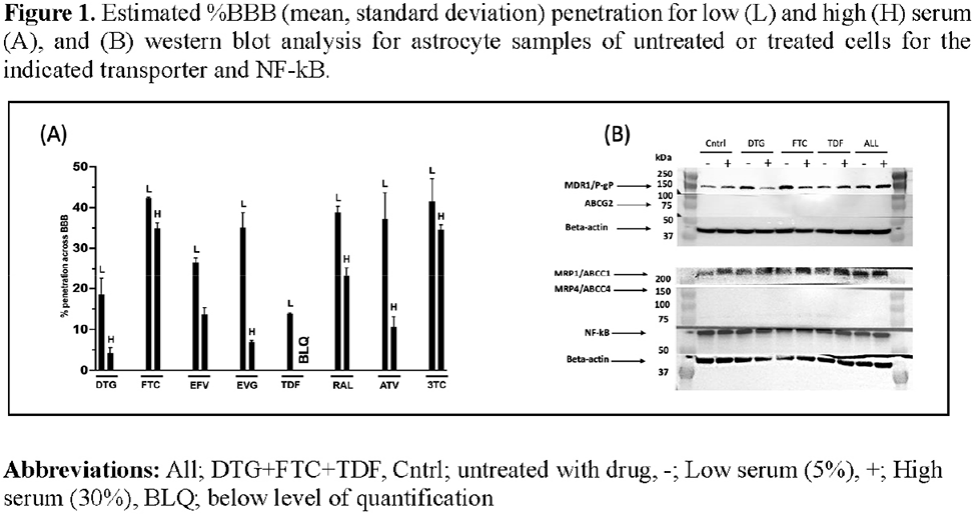

**Methods:**

Two sets of 4-cell (human primary cells) BBB models were evaluated for DTG, FTC, EFV, EVG, TDF, RAL, ATV, and 3TC BBB penetration. In one set, drugs were added with 5% fetal bovine serum (5%FBS; low serum). The other set used 25% FBS + 5% human serum (30% total; high serum). After 24-hours, samples were collected (in triplicate). ARVs were quantitated by validated LC-MS/MS assay. Human astrocyte cells were used for transporter expression determination. After 24-hours of treatment with ARVs, cells were collected and lysed for total protein. Western blot analysis detected ABC transporter and transcription factor NF-kB levels.

**Results:**

The mean %penetration for low serum/high serum in our model, respectively, were: DTG: 25/5; FTC: 40/35; EFV: 27/13; EVG: 30/6; TDF: 14/BLQ; RAL: 38/24; ATV: 37/9; 3TC: 41/34 (Fig 1A). Western blot analysis revealed no difference in the expression of ABCG2 and ABCC4 across drugs or controls (Fig 1B). Exposure to DTG, FTC, TDF, and the combination of all three drugs resulted in greater expression of ABCB1 [i.e., P-glycoprotein (P-gp)] in low serum compared to untreated controls or cells exposed to ARVs in the presence of high serum (Fig 1B). ABCC1 exhibited the opposite pattern of expression level (Fig 1B). NF-kB expression did not differ across treatment or control conditions.

**Conclusion:**

Our novel 4-cell BBB model revealed that ARV %penetration was serum concentration dependent. Our estimates agree with clinical data for low protein binding FTC, EFV, RAL, and 3TC. For high protein binding DTG, EVG, and ATV, our model slightly overestimated clinical results. We found that human astrocytes do not express ABCG2 and ABCC4 but do express P-gp and ABCC1 in the presence of serum and ARVs and that ARVs can induce or inhibit expression of these transporters.

**Disclosures:**

Nathaniel J. Rhodes, PharmD MS, Apothecademy, LLC: Advisor/Consultant

